# Evaluation of the effect of chemical disinfection and ultraviolet disinfection on the dimensional stability of polyether impression material: an in-vitro study

**DOI:** 10.1186/s12903-024-04188-8

**Published:** 2024-04-15

**Authors:** Snehal Joshi, V. N.V Madhav, Ravinder S. Saini, Vishwanath Gurumurthy, Abdulkhaliq Ali F. Alshadidi, Lujain Ibrahim N. Aldosari, Abdulmajeed Okshah, Seyed Ali Mosaddad, Artak Heboyan

**Affiliations:** 1 Department of Prosthodontics, SMBT Dental College and Hospital, Sangamner, 422608 Maharashtra India; 2 Department of Prosthodontics, YCMM & RDF’s Dental College and Hospital, Ahmednagar, India; 3 Department of Dental Technology, COAMS, King Khalid University, Abha, Saudi Arabia; 4 Department of Prosthodontics, College of Dentistry, King Khalid University, Abha, Saudi Arabia; 5 Department of Research Analytics, Saveetha Institute of Medical and Technical Sciences, Saveetha Dental College and Hospitals, Saveetha University, Chennai, India; 6 Student Research Committee, School of Dentistry, Shiraz University of Medical Sciences, Qasr-e-Dasht Street, Shiraz, Iran; 7 Department of Prosthodontics, Faculty of Stomatology, Yerevan State Medical University after Mkhitar Heratsi, Str. Koryun 2, Yerevan, 0025 Armenia; 8 Department of Prosthodontics, School of Dentistry, Tehran University of Medical Sciences, Tehran, Iran

**Keywords:** Polyether impression material, Dimensional stability, Chemical disinfection, Ultraviolet disinfection, In-vitro study, Dental impressions

## Abstract

**Background:**

Various methods, chemical and physical, disinfect dental impressions. Common chemicals include 1% Sodium Hypochlorite and 2% glutaraldehyde, while UV radiation is a prevalent physical method. Few studies compare their effects on dimensional stability in polyether impressions. This study aims to assess such stability using different disinfection methods. Therefore, this study was planned to evaluate the dimensional stability of polyether impression material using different disinfection methods.

**Methods:**

This in vitro study compared the effects of chemical disinfectants (1% Sodium Hypochlorite and 2% glutaraldehyde) and UV irradiation on the dimensional stability of polyether impression material. Groups A, B, C, and D, each with ten samples (*N* = 10), were studied. Group A was untreated (control). Group B was treated with 2% glutaraldehyde for 20 min, Group C with 1% Sodium Hypochlorite for 20 min, and Group D with UV rays for 20 min. A pilot milling machine drill was used to make four parallel holes labeled A, B, C, and D in the anterior and premolar regions from right to left. After sequential drilling, four implant analogs were positioned using a surveyor for accuracy. Ten open-tray polyether impressions were made and treated as described in the groups, followed by pouring the corresponding casts. Distortion values for each disinfection method were measured using a coordinate measuring machine capable of recording on the X- and Y-axes.

**Results:**

A comprehensive analysis was conducted using the one-way ANOVA test for distinct groups labeled A, B, C, and D, revealing significant differences in the mean distances for X1, X2, X4, X5, and X6 among the groups, with *p*-values ranging from 0.001 to 0.000. However, no significant differences were observed in X3. Notably, mean distances for the Y variables exhibited substantial differences among the groups, emphasizing parameter variations, with *p*-values ranging from 0.000 to 0.033. The results compared the four groups using the one-way ANOVA test, revealing statistically significant distance differences for most X and Y variables, except for X3 and Y4. Similarly, post-hoc Tukey’s tests provided specific pairwise comparisons, underlining the distinctions between group C and the others in the mean and deviation distances for various variables on both the X- and Y-axes.

**Conclusions:**

This study found that disinfection with 1% sodium hypochlorite or UV rays for 20 min maintained dimensional stability in polyether impressions.

**Supplementary Information:**

The online version contains supplementary material available at 10.1186/s12903-024-04188-8.

## Background

Patient management, from diagnosis to treatment, relies significantly on dental impressions. Therefore, it is crucial that clinicians understand the properties and manipulation of these impressions. The initial stage involved making an impression. Then, materials such as gypsum are utilized to construct working models. This approach encompasses not only scientific principles but also artistic elements [1].

Polyethers were initially developed in the late 1960s through cationic polymerization. This process involves opening the reactive ethylene imine terminal rings to connect molecules without producing by-products. This material can be effectively used in moist environments owing to its hydrophilic properties. In addition, its commendable wetting properties facilitate the formation of gypsum casts. The most recent versions of polyether impression materials demonstrate a minor increase in flexibility compared to previous versions, which improves their ability to be easily removed from the mouth [2, 3].

Dental implants are structures designed to substitute the root of a lost natural tooth and are commonly employed in rehabilitating individuals with partial or complete tooth loss. Dental implant therapy is widely used to restore patients with missing teeth [4]. Ensuring the precision of the final cast is essential for achieving an accurate impression, which is a critical step in creating properly fitting implant prostheses. A flawed impression can lead to misfit prostheses, potentially causing mechanical and/or biological issues. Although attaining a completely passive fit is challenging, the primary goal in prosthodontic implant procedures is to minimize inconsistencies and decrease the likelihood of complications [5].

To achieve optimal precision, some authors have underscored the significance of interconnecting impression copings through intraoral splinting before the impression-making process. Alternatively, some authors have opted to separate the connection of the splint material, creating a slender gap and subsequently rejoining with a minimal amount of the same material. This approach aims to reduce polymerization shrinkage or involves waiting for complete polymerization of the material to ensure accuracy [6]. Nevertheless, various outcomes have been observed in studies assessing implant impression materials. Researchers have examined the precision of these materials, and polyether has demonstrated superior results compared to condensation silicone, addition silicone, polysulfide, irreversible hydrocolloids, and plaster materials [3, 7].

Dentists routinely create impressions, but these can harbor microorganisms, potentially spreading diseases such as hepatitis B, C, HIV, and tuberculosis. Immediate disinfection after removal from the mouth and clear labeling are essential to prevent contamination and cross-contamination of these impressions. Although rinsing impressions with running water is a common practice to eliminate saliva and blood, it may not effectively eliminate disease-causing microorganisms. Therefore, dentists and dental staff must be familiar with standardized procedures for disinfecting dental impressions and casts. The literature describes various methods of impression disinfection, including chemical disinfection, microwave treatment, autoclaving, and ultraviolet radiation, each with its advantages, disadvantages, and effects on impression materials and casts [8, 9]. Recently, impression materials have been directly integrated with antimicrobials and nanoparticles, enabling the material to possess self-disinfecting properties. This innovation not only ensures the internal disinfection of the impression material but also extends to disinfecting the impressions from the moment they are introduced into the patient’s mouth [10, 11].

Different chemical disinfectants, such as glutaraldehyde and sodium hypochlorite (NaOCl), are used to disinfect impressions. Glutaraldehyde, a pungent colorless solution, can function as a disinfectant in both liquid and gaseous states. Glutaraldehyde, commonly employed to sterilize medical and dental instruments, also serves as a preservative in industrial environments. It possesses potent properties against bacteria, viruses, fungi, spores, and parasites and shows increased effectiveness when organic material concentrations are lower. Properly using these disinfectants requires suitable protective equipment in a well-ventilated environment under the supervision of a trained individual [12].

Sodium hypochlorite, a water-soluble chemical, is widely used in the bleaching and disinfection industries. Its bactericidal activity is attributed to hypochlorous acid (HOCl), a weak acid formed by the addition of water that penetrates microbial cells, inhibits enzyme activity, and damages the cell structure. The concentration of HOCl, influenced by the available chlorine and solution pH, governs its effectiveness as a disinfectant [13].

Various methods of physically disinfecting dental impressions include ultraviolet (UV) and microwave disinfection. The effectiveness of UV rays for disinfection depends on multiple factors, including the duration, intensity, humidity, and accessibility of microorganisms. Because dental prostheses contain numerous sites prone to microorganism growth, UV light must be scattered from multiple angles. Exposure to UV light resulted in a notable decrease in *Candida albicans* colonies compared to low-intensity direct current discharge. Using UV light tubes with higher wattage has been found to significantly reduce colony counts in a shorter time. The most effective antibacterial efficiency through UV light exposure occurs at 24 watts (3750 µw/cm2), and higher wattages result in a faster reduction in C. albicans colony counts, ultimately reaching zero [8, 14]. Samra et al. (2018) proposed that the ultraviolet method is a favorable choice for disinfecting impressions without compromising their dimensional stability [15].

There is a scarcity of studies that have juxtaposed the prevalent chemical disinfection techniques with the physical disinfection method involving UV irradiation, particularly in the context of polyether impression materials. Therefore, this study aimed to evaluate the impact of different disinfection methods on the dimensional stability of polyether impression materials. The objectives of this study were to assess and compare the effect of varying disinfection methods, including chemical disinfection with 2% glutaraldehyde, 1% sodium hypochlorite, and ultraviolet ray disinfection, on the dimensional stability of polyether impression materials. The null hypothesis was that no significant differences would exist between the applied techniques and materials.

## Materials and methods

An in vitro study was conducted to assess the effect of different disinfectants (1% Sodium Hypochlorite and 2% glutaraldehyde) and UV irradiation on the dimensional stability of polyether impression material used in making implant impressions.

### Materials

Four groups were considered and labeled A, B, C, and D in this experimental setting, each comprising ten samples (*N* = 10). Group A served as the control without disinfectant application. In Group B, the samples were treated with glutaraldehyde at a concentration of 2% and subjected to a disinfection time of 20 min. Group C involved the application of 1% NaOCl with a disinfection time of 20 min. In group D, UV rays were used for disinfection, and the exposure time was 20 min. Table 1 outlines the different disinfection methods, concentrations, and durations employed in the experimental design for a comparative analysis of their effectiveness in disinfecting samples. Table 2 also demonstrates all The materials and instruments used in the study.

**Table 1:** Study groups that illustrate different disinfection techniques, disinfectant concentrations, and the corresponding application times

S. No.	GROUP	SAMPLES	DISINFECTANT	CONCENTRATION	TIME
1	A	10	No Disinfectant (Control Group)	---	---
2	B	10	Glutaraldehyde	2%	20 min
3	C	10	Sodium Hypochlorite	1%	20 min
4	D	10	UV	---	20 min

**Table 2:** List of materials and instruments used in the study

Materials	Study Instruments
Gypsum Type IV (Kalrock, Kalabhai, India), 230,802	UV disinfection unit (unicorn DenMart)
Polyethylene vinyl acetate sheet 1.5 mm thick (NMD India), 22,251	Dental Lab Vibrator (De Tax Dental Product),
Modeling wax (MAARC dental wax, Shiva Products, India), 2002/200	Dental vacuum-forming machine (Densply Raintree Essix),
Heat cure acrylic resins (DPI India), 8223	Pentamix machine (3 M ESPE 2)
Tray material (Acralyn-H, Asian Acrylates), 4.07	
Tray adhesive (3 M ESPE), 30,600	
2% Glutaraldehyde (Glutapex), 30,030	
1% sodium hypochlorite. (Molychem), 29,122,990	
Polyether (3 M ESPE Monophase), 4,100,040,082/02	
Adin implant analogs 3.75 × 13 mm (Adin Dental Implant System Israel), 7,516,900	
Impression posts 4.2 mm x 10 mm (Adin Dental Implant System Israel) RS5008	

### Methodology

**Fig. 1:**
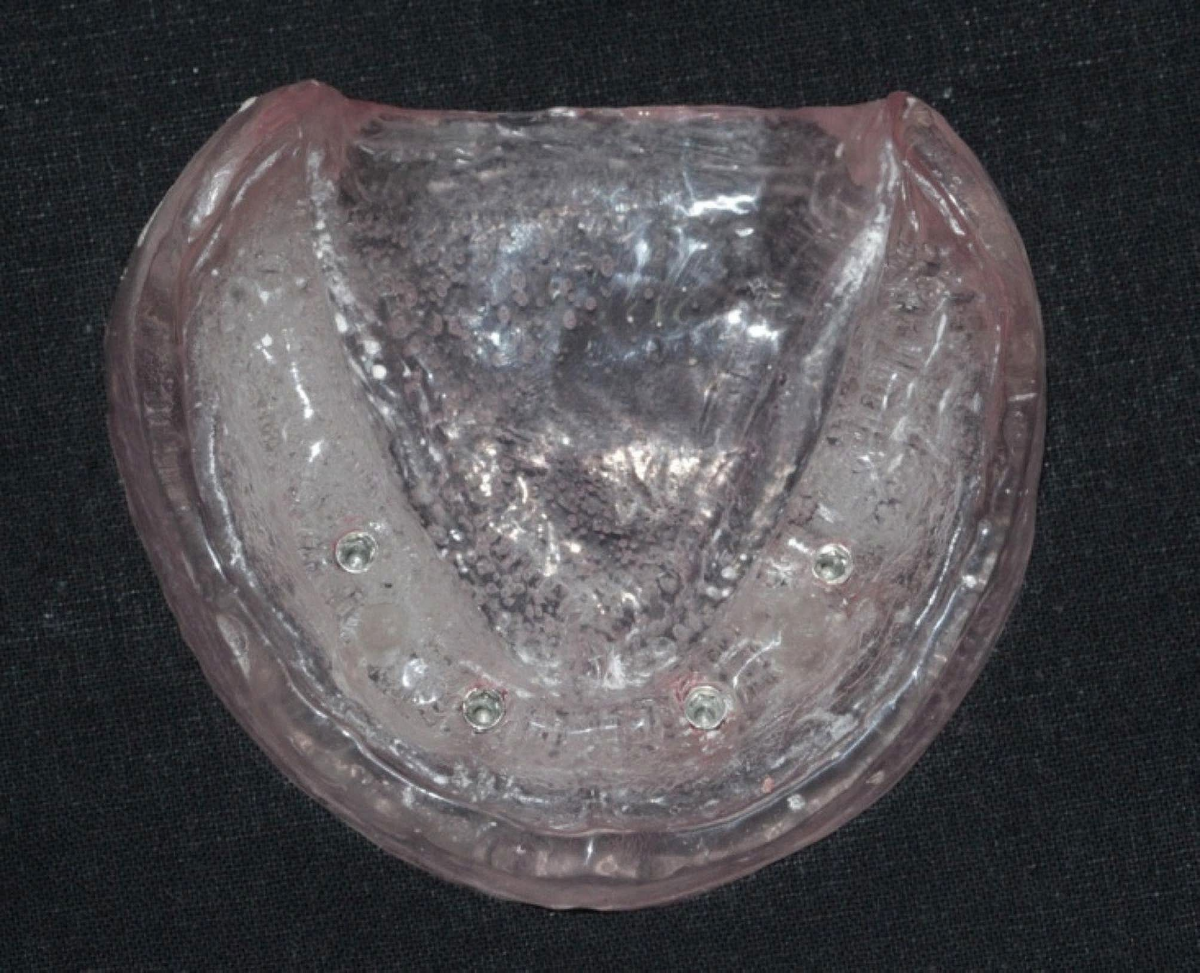
Heat-cured transparent acrylic cast from a master cast wax pattern

**Fig. 2:**
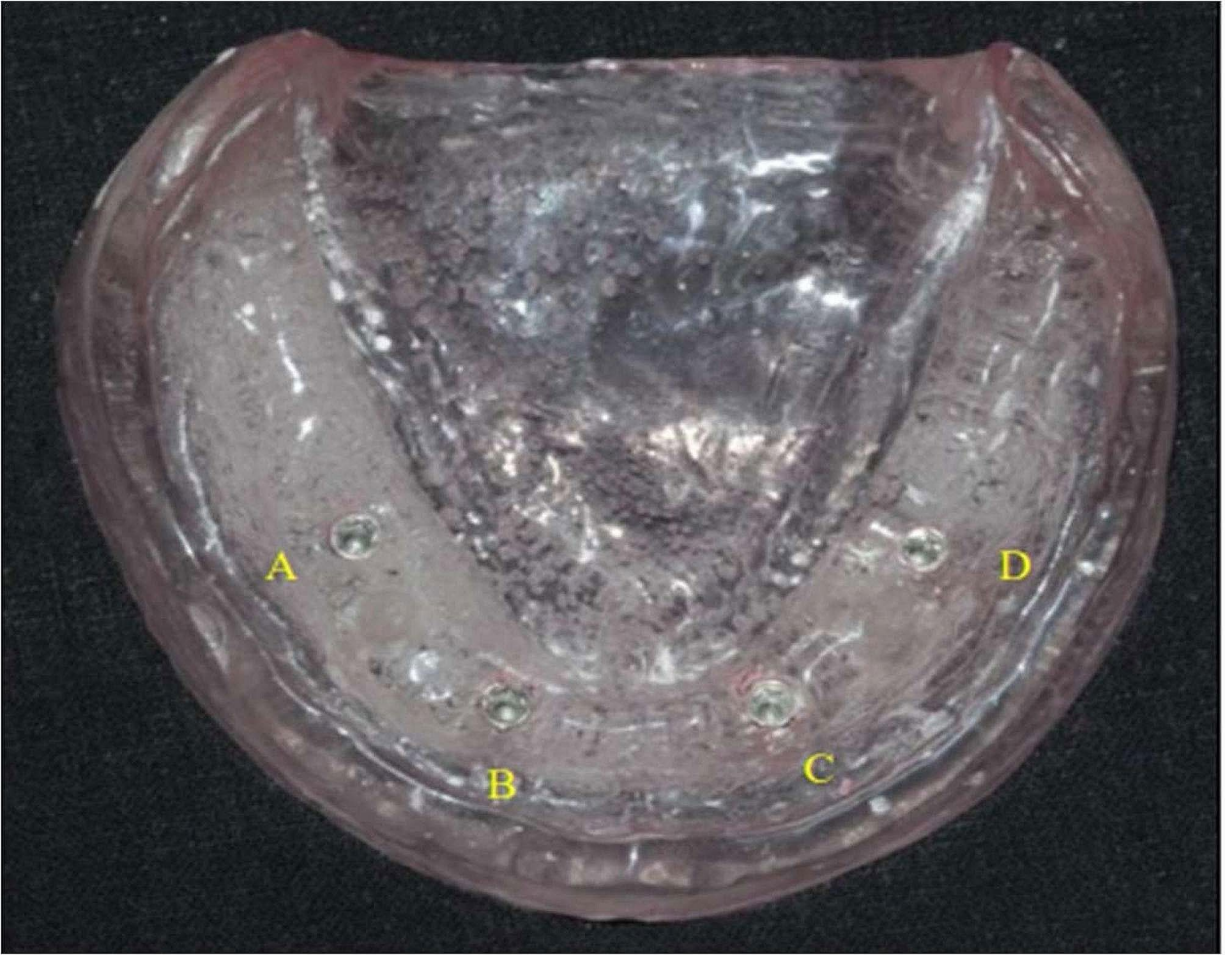
Heat-cured transparent acrylic resin master model with implant analogs numbered A–D from right to left

**Fig. 3:**
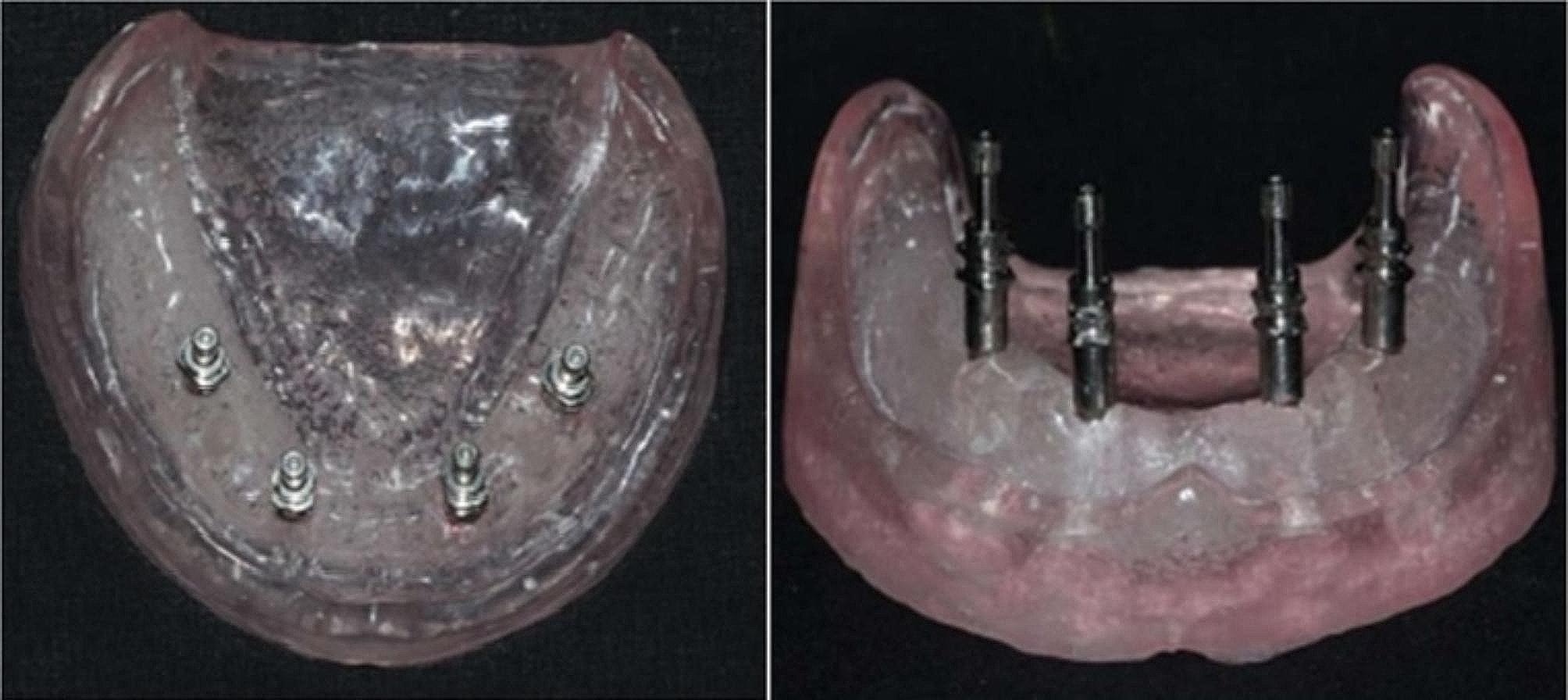
Impression copings affixed to analogs

To construct the model shown in Fig. 1, a premade rubber mold was designed and used to match the edentulous cast index. The melted wax was then poured into a mold, and the master model was fashioned using heat-polymerized clear acrylic resin via heat curing. Figure 2 illustrates the process in the anterior and premolar regions, where a milling machine was employed to create four parallel holes, identified as A, B, C, and D. Sequential drilling was conducted using a pilot drill. Following this, four ADIN implant analogs, featuring a diameter of 3.75 mm and a length of 13 mm with an internal hex, were strategically placed in the acrylic model under the guidance of a surveyor to ensure proper orientation. In Fig. 3, open tray impression posts were manually screwed onto implant analogs, secured with 10-mm flat head guide pins onto the implants using a hex drive, and a torque of 15 N.cm (Newton centimeters) was applied.

### Custom tray fabrication

**Fig. 4:**
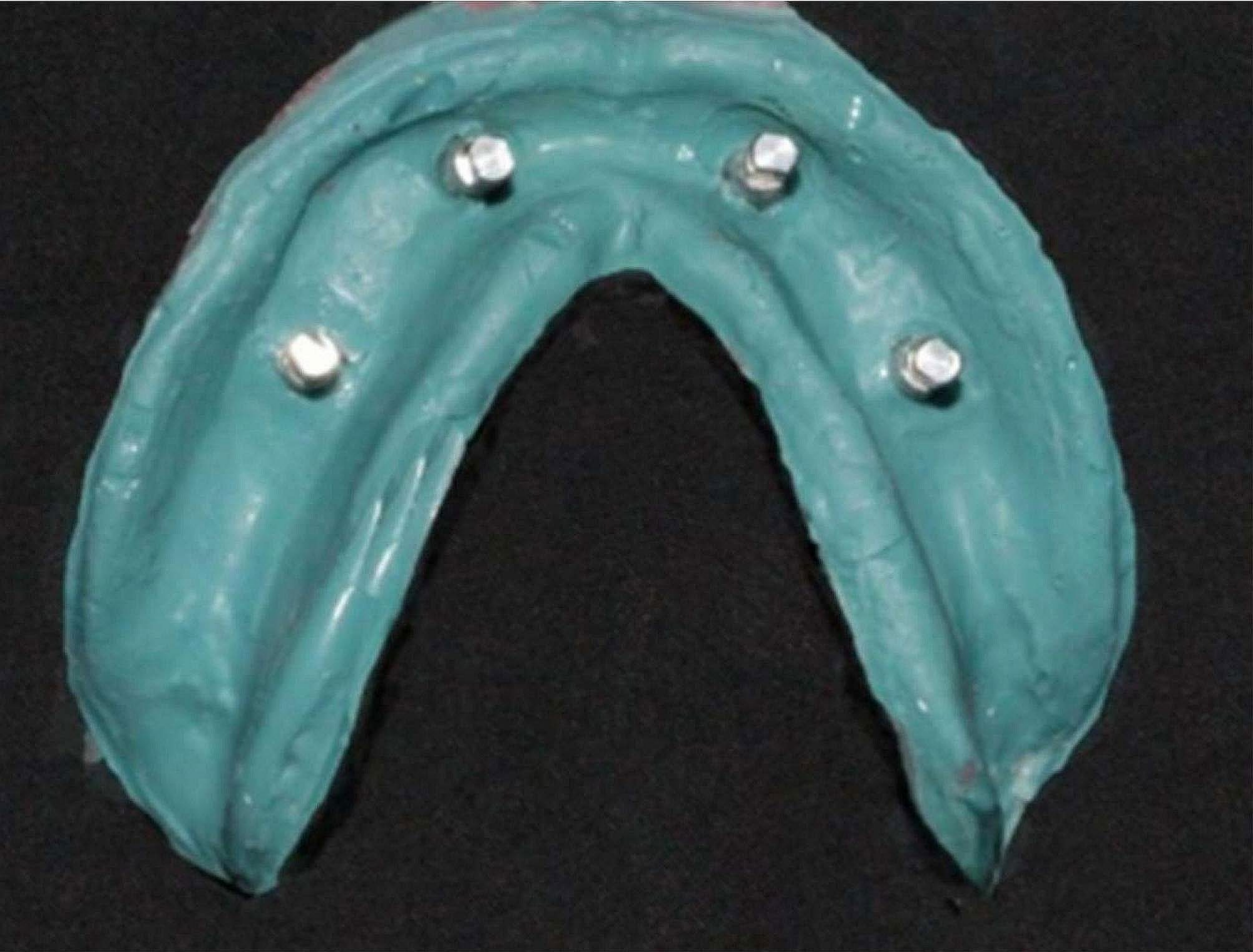
Implant analogs attached to impression copings

Three stoppers, one in the front and two in the back, were placed on the land area of the mandibular master model to ensure proper alignment of the impression trays. A two-layer wax spacer was applied to the master model to create space for the impression material. A custom tray was meticulously fashioned from the tray material to suit the master model (Fig. 4), incorporating windows aligned with the positions of the implant analogs. Forty custom trays were produced, with ten trays per group, each equipped with windows specific to the corresponding region. Subsequently, impressions were made using a medium-body polyether (3 M ESPE Monophase).

### Impression procedure

A custom tray was coated with a tray adhesive, and a machine-mixed medium-body polyether was loaded into the tray with extra material added around the impression posts. The tray was promptly placed on the reference model. Excess material was removed, exposing the guide pins, and maintained during polymerization. After six minutes, the guide pins were unscrewed to separate the tray from the model. The impressions were rinsed, air-dried for 30 min at room temperature, and disinfected. The resulting impressions contained posts and guide pins ready for further dental procedures.

**Fig. 5:**
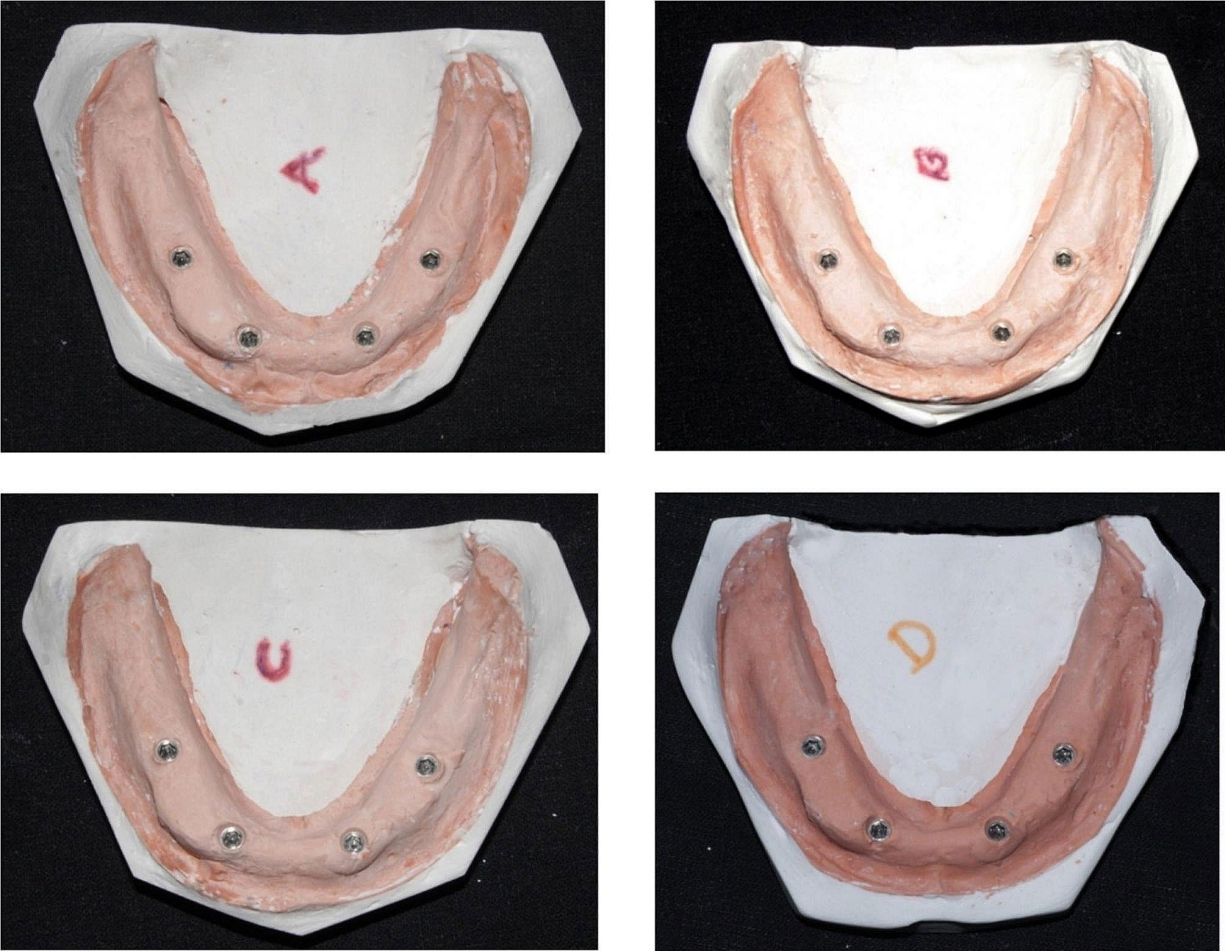
Master casts obtained from open-tray impression copings

As shown in Fig. 5, following disinfection of the impressions, the implant analog was affixed to the impression post, and the guide pins were tightened using a hex driver. Subsequently, the impressions were filled with type-IV gypsum products and allowed to solidify.

### Sampling

**Fig. 6:**
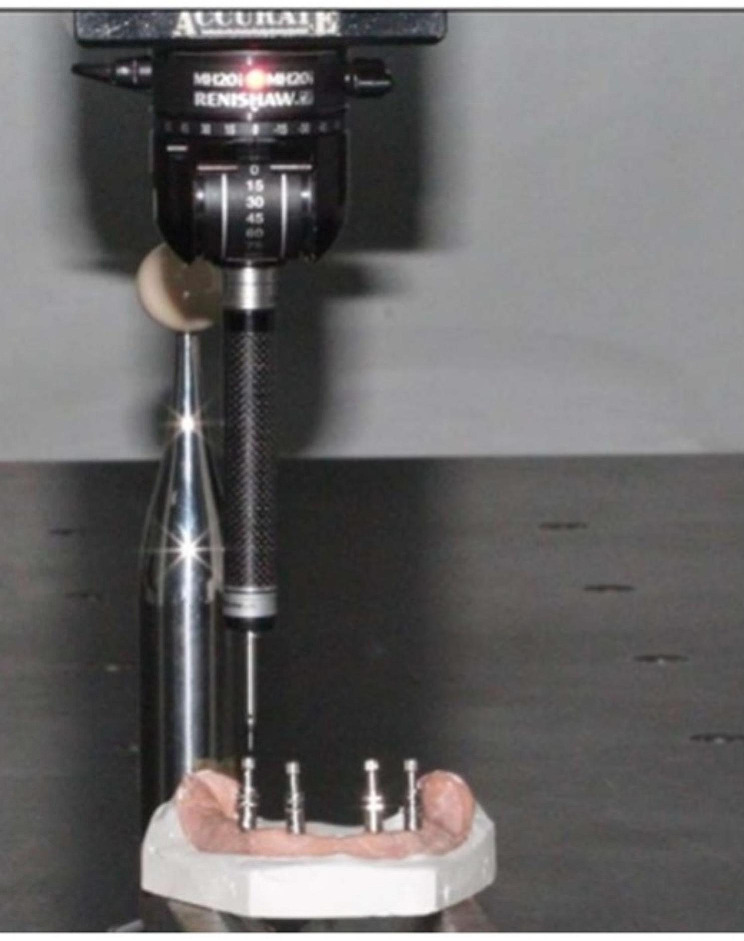
The control model and master distances were measured using a coordinated measurement machine

**Fig. 7:**
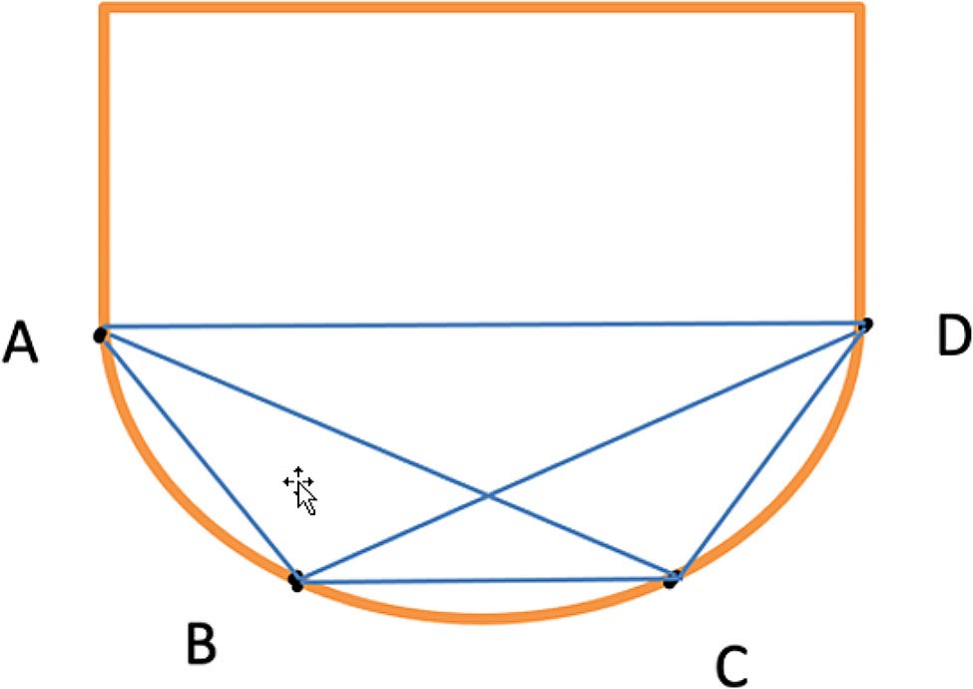
Anteroposterior measurements (AB and CD) and cross-arch measurements (BC, DA, AC, and BD)

As shown in Figure 6, measurements were performed on 40 casts using a coordinated measuring machine. The diameters of the four reference points (A, B, C, and D), each with a known dimension, provided an optimal method for comparing cast measurements with those of the prefabricated model. Points A, B, C, and D on the model were the reference points. Inter-implant distances were measured according to these reference points (Table 3). Figure 7 illustrates anteroposterior measurements (AB and CD) and cross-arch measurements (BC, DA, AC, and BD)

**Table 3:** Inter-implant distances in X Axes and Y-axes

X - Axes		Y - Axes	
Variables	Inter-implant distance	Deviation	Inter-implant distance
X1	A to B	Y1	A to B
X2	C to D	Y2	C to D
X3	B to C	Y3	B to C
X4	D to A	Y4	D to A
X5	A to C	Y5	A to C
X6	B to D	Y6	B to D

### Data management and analysis procedure

The casts were retrieved from each group. The casts were analyzed for the positional accuracy of implant analogs. The data were collected from the coordinate measuring machine on the X- and Y-axes and statistical analysis was performed using the one-way ANOVA test and post hoc tests. Inference was drawn from the obtained data, which are discussed below.

The data were entered into a Microsoft Excel spreadsheet. SPSS software was used for the analysis. Statistical significance was set at *P* < 0.05. was applied. One-way ANOVA was used for statistical data analysis.

## Results

Table 4 presents a comprehensive analysis of mean distances for X1, X2, X3, X4, X5, and X6, as well as Y1, Y2, Y3, Y4, Y5, and Y6, utilizing the one-way ANOVA test. The data were organized into four groups labeled A, B, C, and D, with corresponding subgroup data for X and Y variables, accompanied by mean distances and standard deviations. In the context of the X variables, significant differences were observed in the mean distances for X1, X2, X4, X5, and X6 among Groups A, B, C, and D, indicating group-related effects on these parameters (*p*-values ranging from 0.001to 0.000). However, no significant differences were found for X3. On the other hand, for the Y variables, all mean distances (Y1 to Y6) exhibited significant differences among the groups, suggesting notable variations in these parameters (*p*-values ranging from 0.000 to 0.033). These results collectively underscore the significance of group distinctions in influencing the mean distances for both the X and Y variables, providing valuable insights into the comparative analysis of the studied parameters.

**Table 4:** Comparison of mean distances for X1, X2, X3, X4, X5, and X6 and for Y1, Y2, Y3, Y4, Y5, and Y6 using one-way ANOVA test

	Group No.	A	B	C	D	F	*P*-Value
**Group X (mm)**	1	7.449 ± 0.005	7.334 ± 0.106	7.651 ± 0.143	7.433 ± 0.139	16.546	**0.001**
	2	10.331 ± 0.005	10.292 ± 0.069	10.451 ± 0.104	10.337 ± 0.169	11.845	**0.008**
	3	43.591 ± 0.005	43.421 ± 0.065	43.599 ± 0.124	43.567 ± 0.084	1.408	**0.702**
	4	25.899 ± 0.005	25.691 ± 0.035	25.966 ± 0.215	25.796 ± 0.154	14.808	**0.002**
	5	33.349 ± 0.005	33.121 ± 0.074	33.411 ± 0.071	33.299 ± 0.145	10.209	**0.017**
	6	36.521 ± 0.00033	36.257 ± 0.09808	36.652 ± 0.252	36.451 ± 0.123	19.185	**0.000**
**Group** **Y** **(mm)**	1	14.942 ± 0.005	14.159 ± 0.043	15.871 ± 0.019	14.892 ± 0.061	33.004	**0.000**
	2	14.831 ± 0.005	14.112 ± 0.046	14.982 ± 0.061	14.529 ± 0.152	18.647	**0.000**
	3	0.062 ± 0.006	0.012 ± 0.005	0.091 ± 0.003	0.051 ± 0.004	25.845	**0.000**
	4	0.142 ± 0.004	0.106 ± 0.077	0.191 ± 0.019	0.131 ± 0.109	8.769	**0.033**
	5	14.561 ± 0.008	14.039 ± 0.056	14.653 ± 0.037	14.251 ± 0.129	28.527	**0.000**
	6	14.174 ± 0.005	13.097 ± 0.099	14.273 ± 0.044	13.983 ± 0.045	31.78	**0.000**

All values are in millimeters (mm); F: coefficient of variance

The one-way ANOVA test was employed to compare groups based on various distances (Table 5), namely X1, X2, X4, X5, and X6, with statistically significant differences. However, the results for distance X3 were not statistically significant. Similarly, distances Y1, Y2, Y3, Y4, Y5, and Y6 were statistically significant. In summary, the findings suggest significant distance variations on both the X- and Y-axes among the four groups. Notably, the differences in distances between X3 and Y4 were not statistically significant. This implies that at least one group differs in terms of the distances on these axes.

**Table 5:** Comparison of the four groups using the one-way ANOVA test for distances on the X- and Y-axes

	Group No.	A	B	C	D	X^2^
**MEAN** **Group** **X** **(mm)**	1	18.6	10.3	31.3	21.8	16.546
	2	12.6	25.2	16.1	28.1	11.845
	3	19.5	23.5	17.6	21.4	1.408
	4	19.5	31.4	11.5	19.6	14.808
	5	29.5	18.7	20.7	13.1	10.209
	6	21.5	32.7	10.5	17.3	19.185
**MEAN** **Groups ** **Y** **(mm)**	1	5.5	20.3	35.5	20.7	33.004
	2	9.5	30.7	24.7	17.1	18.647
	3	5.5	31.2	21.3	24	25.845
	4	29.5	15.4	17	20.1	8.769
	5	9.5	35.5	22.7	14.3	28.527
	6	5.5	18.1	34.3	24.1	31.78

All values are in millimeters (mm); X2 is the chi-square test coefficient

### Pairwise comparison for mean distances

#### Post hoc tests

Table 6 displays the results of a post-hoc test conducted for pairwise comparisons using Tukey’s test. Statistically significant results were observed for distance X1 in comparisons between groups C and A, groups C and 2, and groups C and D. Similarly, significant findings were noted for distance X4 in comparisons between groups C and A, groups C and B, and groups C and D. For distance X5, significant differences were observed between groups C and A, groups C and B, and groups C and D. Significant results were also obtained for distance X6 in comparisons between groups C and A, groups C and B, and groups C and D. Conversely, all other comparisons yielded non-significant results. This pattern was repeated for distances Y1, Y4, Y5, and Y6, with significant findings in specific group C comparisons and non-significant results in the others.

**Table 6:** Significant Pairwise comparison of post-hoc Tukey’s Test for X-Y distances (mm)-only significant results are shown

Dependent Variable	(I) GROUPS	(J) GROUPS	Mean Difference (I- J)	*P* value	Dependent Variable	(I) GROUPS	(J) GROUPS	Mean Difference (I- J)	*P* value
MEAN X1	A	C	− 0.2028400*	0.002**	MEAN Y1	A	C	− 0.3765500*	001**
	B	C	− 0.3177400*	0.000**		B	C	− 0.0902500*	0.000**
	C	A	0.2028400*	0.002**		C	A	0.3765500*	0.001**
		B	0.3177400*	0.000**			B	0.0902500*	0.000**
		D	0.2182500*	0.001**			D	0.0958700*	0.001**
	D	C	− 0.2182500*	0.001**		D	C	− 0.0958700*	0.001**
MEAN X4	A	C	0.2257600*	0.003**	MEAN Y4	A	C	0.0324100*	0.006**
	B	C	0.2925900	0.000**		B	C	− 0.0036900	0.000**
	C	A	− 0.2257600	0.003**		C	A	− 0.0324100*	0.006**
		B	− 0.2925900	0.000**			B	0.0036900*	0.000**
		D	− 0.1229000	0.001**			D	− 0.0197200	0.001**
	D	C	0.1229000	0.001**		D	C	0.0197200*	0.001**
MEAN X5	A	C	0.0260500	0.026*	MEAN Y5	A	C	− 0.1240900	0.029*
	B	C	− 0.0233900	0.023*		B	C	0.1350700*	0.024*
	C	A	− 0.0260500	0.026*		C	A	0.1240900*	0.029*
		B	0.0233900	0.023*			B	− 0.1350700	0.024*
		D	0.0925500	0.031*			D	0.1001700*	0.041*
	D	C	− 0.0925500	0.031*		D	C	− 0.1001700	0.041*
MEAN X6	A	C	0.2365600*	006**	MEAN Y6	A	C	− 0.3986400*	0.002**
	B	C	0.3705200*	000**		B	C	− 0.1757600*	0.000**
	C	A	− 0.2365600*	006**		C	A	0.3986400*	0.002**
		B	− 0.3705200*	000**			B	0.1757600*	0.000**
		D	− 0.2465500*	004**			D	0.0693200*	0.001**
	D	C	0.2465500*	004**		D	C	− 0.0693200*	0.001**

Note: Only significant results are shown

## Discussion

In this study, the effects of chemical and UV disinfection on the dimensional stability of polyether impression materials was evaluated. Based on the results of the study, it is suggested that applying different disinfection methods (chemical and UV) has a discernible influence on these dimensions. However, no significant differences were found, indicating that specific dimensions may be less susceptible to variations induced by disinfection methods. Similarly, all mean distances exhibited substantial differences among the groups for the Y variables, representing another set of dimensional parameters. This highlights that the dimensional stability of the polyether impression material is significantly affected by both chemical and UV disinfection, impacting various aspects (denoted by Y1–Y6).

Previous studies have demonstrated using chemical disinfectants, including glutaraldehyde, NaOCl, Dettol, Silosept, and Cavex, to disinfect impression materials, such as polyvinyl ether siloxane (PVES). Immersing PVES impressions in chemical disinfectants for 10 min led to clinically irrelevant changes in dimensions. However, the choice of disinfectant type and chemical nature may lead to water imbibition, potentially compromising accuracy [16]. In the study conducted by Nimonkar et al., the effects of chemical and UV disinfectants on the dimensional stability of polyvinyl siloxane impressions were investigated; the results revealed that immersion in chemical disinfectants significantly influenced dimensional accuracy, whereas UV disinfection emerged as a simple and effective method. Additionally, UV radiation has proven beneficial in safeguarding materials from the potential harm caused by chemical exposure [17]. Walker et al. observed alterations in the dimensions of vinyl polysiloxane and polyether after exposure to 0.5% NaOCl or phenol disinfectants for 10 min and one hour [18]. These findings are crucial for dental professionals and suggest that carefully considering disinfection protocols is essential to maintain the accuracy and reliability of impressions obtained using polyether materials in clinical settings. Studies on the effectiveness of commercial household UV-C germicidal devices in Thailand found that UVC irradiance and distance play crucial roles in the efficacy of surface disinfection. This study also highlights the importance of proper application and the short effective range of UVC irradiance, typically less than 10 cm [17].

The results further revealed significant variations in the mean distances between the tested groups for both the X- and Y-axis measurements. In terms of chemical disinfection, Groups B, C, and D, treated with Glutaraldehyde, NaOCl, and UV rays, respectively, displayed distinct mean distances compared to the Control Group (Group A) and shows that notably, the application of Glutaraldehyde, NaOCl, and UV rays may introduce alterations in the dimensional characteristics of the material. The variations in the mean distances observed in both the X- and Y-axis measurements indicate potential effects on the accuracy and reliability of impressions when subjected to these disinfection methods.

The results also revealed that for the X-axis, the statistical analysis indicated notable differences in the mean values between different disinfection methods. Specifically, the mean values for groups subjected to glutaraldehyde (Group B), NaOCl (Group C), and UV rays (Group D) differed significantly from those of the control Group A (group A) at multiple data points (1, 2, 4, 5, and 6). The stability of the polyether impression material appeared to be affected by the disinfection method used, as is evident from the variations in the mean values on the Y-axis. These differences across the data points highlight the significant impact of disinfection choice on the material’s dimensional stability. These results emphasize the crucial role of disinfection techniques in preserving the accuracy of polyether impressions. Aeran et al. assessed the efficacy of UV-C radiation in disinfecting alginate, A-silicone, and polyether impression materials [19]. Research has revealed that materials such as polyether, hydrocolloid, C-silicone, and A-silicone experience dimensional changes within clinically acceptable levels when disinfected with either 1% NaOCl or an aldehyde-free disinfectant solution, as per American Dental Association standards [14]. Shetty et al. conducted a thorough investigation of the impact of immersion disinfection on the wettability of polyether impression materials. This study revealed significant alterations in the decontamination of impression surfaces owing to the disinfection procedure. Consequently, changes in the wettability of polyether impression materials have been observed [20]. Notably, UV disinfection, glutaraldehyde chemical disinfection, and NaOCl had distinct effects on dimensional stability compared to the control group. The statistical analysis conducted in this study indicated significant differences in polyether impressions. Among the three disinfecting materials, 2% Glutaraldehyde and UV rays showed the lowest dimensional change. The results of the present study show that disinfection with 2% glutaraldehyde and UV rays can be used as an alternative disinfection method for polyether impression materials.

Furthermore, the results demonstrated noteworthy variations among the four groups (A, B, C, and D) regarding their dimensional stability. A highly significant difference (*P* < 0.01) was observed among the groups in the X-axis analysis. This indicates that the dimensional stability of polyether impressions differed significantly between the control group (no disinfectant) and the groups subjected to chemical or UV disinfection. Similarly, a significant difference (*P* = 0) emerged between the groups in the Y-axis analysis, indicating that at least one group exhibited considerable deviation from the others in terms of dimensional stability along the Y-axis. These results suggest that selecting a chemical or UV-based disinfection method significantly influences the dimensional stability of polyether impressions. This highlights the importance of choosing appropriate disinfection methods to maintain the integrity of polyether impressions.

In the examination of distances along the x-axis, significant variations emerged in pairwise comparisons involving Group C. Distances X1, X4, X5, and X6 exhibited statistically significant differences when compared to Groups A, B, and D. These findings suggest that the dimensional stability of the polyether impression material in Group C was influenced by the disinfection method employed, leading to specific variations in distance. It is crucial to highlight that non-significant differences were observed in the remaining comparisons, indicating a similarity in dimensional stability among the groups. Likewise, in the analysis of distances along the Y-axis, Group C demonstrated significant disparities in distances Y1, Y4, Y5, and Y6 when compared to Groups A, B, and D. This implies that the choice of disinfection method affected the dimensional stability of the polyether impression material at these distances along the Y-axis. Again, non-significant differences were noted in the remaining comparisons, indicating comparable dimensional stability among the groups. A study conducted by Williams et al. explored the linear dimensional accuracy of three elastomeric impression materials (polyether, addition silicone, and condensation silicone) at various time intervals. The results indicated that polyether exhibited outstanding dimensional stability and the lowest degree of shrinkage [21]. This research demonstrates that the chemical disinfection method used in Group C had a significant impact on the dimensional stability of the polyether impression material at specific distances along the X- and Y-axes. These results offer important insights into the efficacy of disinfection methods in preserving the dimensional stability of dental impression materials, thereby enhancing our understanding of optimal dental procedures.

The analysis results, which considered deviation distances along both the X- and Y-axes, revealed statistically significant variances in the deviation distances for specific comparisons. Specifically, in the X-axis, group C (treated with NaOCl) showed significant differences compared to groups A, B, and D for several deviation distances (X1, X4, X5, and X6). However, the other comparisons were not statistically significant (*p* > 0.05). Similarly, on the Y-axis, group C exhibited substantial differences from groups A, B, and D for certain deviation distances (Y1, Y4, Y5, and Y6), whereas the other comparisons were non-significant. This study suggests that the choice of disinfection method, specifically NaOCl in this case, may impact the dimensional stability of polyether impression materials in certain situations. However, this also indicates that there are instances in which the choice of disinfection method may not have a significant effect. Overall, this study provides valuable insights into how different disinfection methods affect the dimensional stability of polyether impression materials, highlighting specific scenarios where notable differences were observed. Various techniques, such as immersion and spraying, have proven effective in disinfecting impression material surfaces at varying concentrations and application times. Numerous studies have explored the impact of different disinfectants on the dimensional stability of various dental impression materials, including polyether [22]. The null hypothesis was therefore accepted as no significant differences were found with the applied techniques and materials.

The study had various limitations, as follows:

1. The study was performed in vitro, which differs from the clinical scenario, and factors related to clinical settings were not considered.
2. The study included only polyether impression materials; other materials and techniques were not investigated.
3. A single non-blinded investigator performed all laboratory procedures, which could have led to errors and bias.

In conclusion, this in vitro study offers valuable insights into the effects of chemical and UV disinfection on the dimensional stability of polyether impression materials. These findings contribute to a broader understanding of disinfection protocols in dental procedures and may inform practitioners regarding considerations for maintaining the accuracy of impressions in clinical settings. Further research and clinical validation may be necessary to contextualize these findings within the broader scope of dental practice.

## Conclusion

The following conclusions were drawn within the limitations of the study.

1. Impressions using polyether consistently produced master casts with measurements closely aligned to the reference model, which remained within clinical limits.
2. Among the three disinfection methods, chemical disinfection (1% sodium hypochlorite immersion) and physical disinfection (20-minute exposure to UV rays) proved more reliable than the other disinfection methods.
3. The dimensional changes observed in the polyether impression material specimens were within the acceptable range of the maximum linear dimensional changes (%) recommended by ISO 4823.

### Electronic supplementary material

Below is the link to the electronic supplementary material.

Supplementary Material 1

## Data Availability

The data supporting this study’s findings are available from the corresponding author upon reasonable request.

## Abbreviations

NaOCl: Sodium hypochlorite
HOCl: Hypochlorous acid
UV: Ultraviolet
PVES: Polyvinyl ether siloxane

## References

[1] Gupta R, Brizuela M. Dental Impression Materials. StatPearls. 2023. 34662010

[2] Pandey P, Mantri S, Bhasin A, Deogade S (2019). Mechanical properties of a New Vinyl Polyether silicone in comparison to Vinyl Polysiloxane and Polyether Elastomeric impression materials. Contemp Clin Dent.

[3] Awod Bin Hassan S, Ali FAA, Ibrahim NAL, Heboyan A (2023). Effect of chemical disinfection on the dimensional stability of polyvinyl ether siloxane impression material: a systemic review and meta-analysis. BMC Oral Health.

[4] Yasar MN, Cetinsahin C, Bayar O, Ozer HY. Implant Impression techniques using different materials and methods: a review. Journal of clinical and diagnostic research; 2022.

[5] Montero J. A review of the major prosthetic factors influencing the prognosis of Implant Prosthodontics. J Clin Med. 2021;10(4). 10.3390/jcm10040816 PMC7921991 33671394

[6] Patil P, Madhav VNV, Alshadidi AAF, Saini RS, Aldosari LIN, Heboyan A (2023). Comparative evaluation of open tray impression technique: investigating the precision of four splinting materials in multiple implants. BMC Oral Health.

[7] Zarrintaj P, Rezaei S, Jafari SH, Saeb MR, Ghalami S, Roshandel M et al. Impression materials for dental prosthesis. Adv Dent Biomaterials. 2019:197–215.

[8] Kotwal M, Singh V, Mushtaq H, Ahmed R, Rai G, Kumar A (2021). Disinfection of impression materials with glutaraldehyde, Ultraviolet Radiation, and autoclave: a comparative study. J Pharm Bioallied Sci.

[9] Heboyan A, Bennardo F (2023). New biomaterials for modern dentistry. BMC Oral Health.

[10] Al Mortadi N, Al-Khatib A, Alzoubi KH, Khabour OF (2019). Disinfection of dental impressions: knowledge and practice among dental technicians. Clin Cosmet Invest Dentistry.

[11] Saini RS, Alshadidi AAF, Hassan SAB, Aldosari LIN, Mosaddad SA, Heboyan A (2024). Properties of a novel composite elastomeric polymer vinyl polyether siloxane in comparison to its parent materials: a systemic review and meta-analysis. BMC Oral Health.

[12] Chidambaranathan AS, Balasubramanium M (2019). Comprehensive Review and comparison of the disinfection techniques currently available in the literature. J Prosthodont.

[13] Sodium Hypochlorite. | NaClO | CID 23665760 - PubChem. 2023.

[14] Wezgowiec J, Paradowska-Stolarz A, Malysa A, Orzeszek S, Seweryn P, Wieckiewicz M. Effects of various disinfection methods on the Material properties of Silicone Dental impressions of different types and viscosities. Int J Mol Sci. 2022;23(18). 10.3390/ijms231810859 PMC9505442 36142778

[15] Samra RK, Bhide SV (2018). Comparative evaluation of dimensional stability of impression materials from developing countries and developed countries after disinfection with different immersion disinfectant systems and ultraviolet chamber. Saudi Dent J.

[16] Awod Bin Hassan S, Ali F, Alshadidi A, Ibrahim N, Aldosari L, Heboyan A, Saini S. R. Effect of chemical disinfection on the dimensional stability of polyvinyl ether siloxane impression material: a systemic review and meta-analysis. BMC Oral Health. 2023;23(1). 10.1186/s12903-023-03168-8 PMC10331969 37430254

[17] Nimonkar SV, Belkhode VM, Godbole SR, Nimonkar PV, Dahane T, Sathe S (2019). Comparative evaluation of the Effect of Chemical disinfectants and Ultraviolet Disinfection on Dimensional Stability of the polyvinyl siloxane impressions. J Int Soc Prev Community Dentistry.

[18] AlZain S (2020). Effect of chemical, microwave irradiation, steam autoclave, ultraviolet light radiation, ozone and electrolyzed oxidizing water disinfection on properties of impression materials: a systematic review and meta-analysis study. Saudi Dent J.

[19] Vrbova R, Bradna P, Bartos M, Roubickova A (2020). The effect of disinfectants on the accuracy, quality and surface structure of impression materials and gypsum casts: a comparative study using light microscopy, scanning electron microscopy and micro computed tomography. Dent Mater J.

[20] Hardan L, Bourgi R, Cuevas-Suárez CE, Lukomska-Szymanska M, Cornejo-Ríos E, Tosco V (2022). Disinfection procedures and their effect on the Microorganism colonization of Dental Impression materials: a systematic review and Meta-analysis of in Vitro studies. Bioengineering.

[21] Khan SA, Tushar, Nezam S, Singh P, Kumari N, Singh SS (2020). Comparison and evaluation of Linear Dimensional Accuracy of three elastomeric impression materials at different time intervals using Vision Inspection System: an in Vitro Study. J Int Soc Prev Community Dentistry.

[22] Wezgowiec J, Wieczynska A, Wieckiewicz M, Czarny A, Malysa A, Seweryn P et al. Evaluation of Antimicrobial Efficacy of UVC Radiation, Gaseous ozone, and Liquid Chemicals used for disinfection of Silicone Dental Impression materials. Materials. 2022;15(7). 10.3390/ma15072553 PMC8999620 35407884

