# Supplementary material for: Evaluation of the effect of chemical disinfection and ultraviolet disinfection on the dimensional stability of polyether impression material: an in-vitro study
Source: BMC Oral Health. 2024 Apr 15;24:458. doi: 10.1186/s12903-024-04188-8 (PMC11020824; doi:10.1186/s12903-024-04188-8)
Supplement: Supplementary file 1 — Supplementary Material 1 [file 12903_2024_4188_MOESM1_ESM.docx]

Measurements obtained from Co-Ordinated Measuring Machine on X and Y axis

| S.NO | GROUPS | MEAN  X1 | DEVI X1 | MEAN X2 | DEVI X2 | MEAN X3 | DEVI X3 | MEAN X4 | DEVI X4 | MEAN X5 | DEVI X5 | MEAN X6 | DEVI X6 |
| --- | --- | --- | --- | --- | --- | --- | --- | --- | --- | --- | --- | --- | --- |
| 1 | 1 | 7.449 | 0.251 | 10.2245 | 0.246 | 43.5734 | 0.184 | 25.899 | 25.899 | 0.121 | 0.284 | 36.124 | 0.115 |
| 2 | 1 | 7.334 | 7.334 | 7.334 | 7.334 | 7.334 | 7.334 | 7.334 | 7.334 | 7.334 | 7.334 | 7.334 | 7.334 |
| 3 | 1 | 7.65 | 0.354 | 10.451 | 0.296 | 43.499 | 0.191 | 25.966 | 25.966 | 0.229 | 0.296 | 36.652 | 0.219 |
| 4 | 1 | 7.4 | 0.236 | 10.337 | 0.232 | 43.567 | 0.181 | 25.796 | 25.796 | 0.085 | 0.193 | 36.451 | 0.091 |
| 5 | 1 | 7.8 | 0.115 | 10.251 | 0.632 | 43.652 | 0.151 | 23.961 | 25.361 | 0.089 | 0.785 | 36.154 | 0.019 |
| 6 | 1 | 7.6 | 0.235 | 10.523 | 0.325 | 43.123 | 0.225 | 21.853 | 23.865 | 0.045 | 0.986 | 36.514 | 0.015 |
| 7 | 1 | 7.55 | 0.365 | 10.365 | 0.365 | 42.125 | 0.365 | 22.894 | 24.369 | 0.091 | 0.327 | 35.961 | 0.189 |
| 8 | 1 | 7.25 | 0.52 | 10.215 | 0.145 | 44.652 | 0.528 | 23.891 | 25.619 | 0.0884 | 0.329 | 36.875 | 0.364 |
| 9 | 1 | 7.89 | 0.036 | 10.658 | 0.254 | 43.256 | 0.635 | 25.632 | 23.859 | 0.556 | 0.945 | 34.326 | 0.985 |
| 10 | 1 | 7.35 | 0.113 | 10.352 | 0.365 | 43.852 | 0.368 | 23.987 | 24.967 | 0.337 | 0.664 | 36.789 | 0.327 |

| SLNO | GROUPS | MEAN  X1 | DEVI X1 | MEAN X2 | DEVI X2 | MEAN X3 | DEVI X3 | MEAN X4 | DEVI X4 | MEAN X5 | DEVI X5 | MEAN X6 | DEVI X6 |
| --- | --- | --- | --- | --- | --- | --- | --- | --- | --- | --- | --- | --- | --- |
| 1 | 2 | 7.334 | 7.334 | 7.334 | 7.334 | 7.334 | 7.334 | 7.334 | 7.334 | 7.334 | 7.334 | 7.334 | 7.334 |
| 2 | 2 | 0.099 | 0.099 | 0.251 | 0.6 | 43.59 | 0.005 | 0.0064 | 0.251 | 6.625 | 0.678 | 7.332 | 7.653 |
| 3 | 2 | 0.015 | 0.043 | 0.136 | 0.069 | 43.42 | 0.065 | 0.0054 | 0.046 | 7.562 | 0.87 | 7.297 | 7.453 |
| 4 | 2 | 0.087 | 0.021 | 0.354 | 0.104 | 43.59 | 0.124 | 0.85 | 0.061 | 7.62 | 0.098 | 6.098 | 7.54 |
| 5 | 2 | 0.085 | 0.012 | 0.652 | 0.235 | 43.65 | 0.125 | 0.78 | 0.051 | 7.612 | 0.091 | 6.021 | 7.459 |
| 6 | 2 | 0.012 | 0.018 | 0.326 | 0.366 | 43.56 | 0.124 | 0.36 | 0.045 | 7.361 | 0.095 | 7.659 | 7.856 |
| 7 | 2 | 0.217 | 0.026 | 0.652 | 0.544 | 43.98 | 0.325 | 0.98 | 0.065 | 7.586 | 0.096 | 6.256 | 7.325 |
| 8 | 2 | 0.035 | 0.056 | 0.321 | 0.859 | 43.89 | 0.125 | 0.48 | 0.078 | 7.451 | 0.093 | 6.327 | 7.961 |
| 9 | 2 | 0.052 | 0.065 | 0.315 | 0.366 | 43.65 | 0.126 | 0.95 | 0.89 | 7.621 | 0.095 | 7.329 | 7.324 |
| 10 | 2 | 0.065 | 0.084 | 0.895 | 0.988 | 42.32 | 0.124 | 0.62 | 0.045 | 7.361 | 0.097 | 7.659 | 7.361 |

| SLNO | GROUPS | MEAN  X1 | DEVI X1 | MEAN X2 | DEVI X2 | MEAN X3 | DEVI X3 | MEAN X4 | DEVI X4 | MEAN X5 | DEVI X5 | MEAN X6 | DEVI X6 |
| --- | --- | --- | --- | --- | --- | --- | --- | --- | --- | --- | --- | --- | --- |
| 1 | 3 | 7.65 | 0.354 | 10.451 | 0.296 | 43.499 | 0.191 | 25.966 | 25.966 | 0.229 | 0.296 | 36.652 | 0.219 |
| 2 | 3 | 0.09 | 14.56 | 9.89 | 0.009 | 54.76 | 0.009 | 6.987 | 0.007 | 0.09 | 1.09 | 35.664 | 0.213 |
| 3 | 3 | 0.136 | 0.23 | 10.87 | 0.098 | 0.098 | 0.321 | 0.098 | 0.432 | 0.08 | 1.76 | 32.008 | 0.233 |
| 4 | 3 | 0.345 | 0.543 | 0.786 | 7.76 | 0.07 | 16.78 | 0.907 | 0.12 | 0.09 | 34.76 | 0.652 | 0.231 |
| 5 | 3 | 0.025 | 0.352 | 0.366 | 0.003 | 0.089 | 0.012 | 0.805 | 0.352 | 0.07 | 1.62 | 0.638 | 0.265 |
| 6 | 3 | 0.125 | 0.253 | 0.788 | 0.006 | 0.012 | 0.563 | 0.521 | 0.688 | 0.045 | 1.35 | 0.648 | 0.124 |
| 7 | 3 | 0.135 | 0.635 | 0.985 | 0.0325 | 0.035 | 0.986 | 0.987 | 0.955 | 0.094 | 0.321 | 0.691 | 0.125 |
| 8 | 3 | 0.06 | 0.123 | 0.477 | 0.021 | 0.063 | 1.326 | 0.635 | 0.961 | 0.015 | 0.854 | 0.631 | 0.129 |
| 9 | 3 | 0.008 | 0.321 | 0.545 | 0.065 | 0.085 | 0.326 | 0.339 | 0.331 | 0.065 | 0.961 | 0.259 | 0.126 |
| 10 | 3 | 0.485 | 0.231 | 0.852 | 0.986 | 0.36 | 0.256 | 0.332 | 0.257 | 0.067 | 0.655 | 0.784 | 0.126 |

| SLNO | GROUPS | MEAN  X1 | DEVI X1 | MEAN X2 | DEVI X2 | MEAN X3 | DEVI X3 | MEAN X4 | DEVI X4 | MEAN X5 | DEVI X5 | MEAN X6 | DEVI X6 |
| --- | --- | --- | --- | --- | --- | --- | --- | --- | --- | --- | --- | --- | --- |
| 1 | 4 | 7.4 | 0.236 | 10.337 | 0.232 | 43.567 | 0.181 | 25.796 | 25.796 | 0.085 | 0.193 | 36.451 | 0.091 |
| 2 | 4 | 0.09 | 0.345 | 0.098 | 0.231 | 0.89 | 0.003 | 23.789 | 21.76 | 0.076 | 0.871 | 1.054 | 0.091 |
| 3 | 4 | 0.87 | 0.043 | 0.776 | 0.078 | 1.009 | 1.009 | 23.987 | 23.876 | 0.098 | 0.181 | 0.087 | 0.098 |
| 4 | 4 | 1.98 | 1.78 | 1.097 | 0.891 | 0.008 | 0.009 | 21.897 | 22.786 | 0.9 | 0.094 | 0.564 | 0.012 |
| 5 | 4 | 0.05 | 0.564 | 1.321 | 0.652 | 0.005 | 0.009 | 22.532 | 22.945 | 0.975 | 0.098 | 0.567 | 0.014 |
| 6 | 4 | 0.33 | 0.654 | 0.698 | 0.326 | 0.004 | 0.012 | 25.897 | 23.756 | 0.948 | 0.095 | 0.561 | 0.016 |
| 7 | 4 | 0.6 | 0.456 | 0.658 | 0.357 | 0.001 | 0.036 | 21.632 | 24.634 | 0.962 | 0.094 | 0.555 | 0.019 |
| 8 | 4 | 0.65 | 0.963 | 0.365 | 0.658 | 0.008 | 0.096 | 23.627 | 21.652 | 0.932 | 0.096 | 0.561 | 0.015 |
| 9 | 4 | 0.335 | 0.369 | 0.458 | 0.654 | 1.036 | 0.075 | 22.631 | 23.458 | 0.937 | 0.092 | 0.562 | 0.013 |
| 10 | 4 | 0.95 | 0.635 | 0.457 | 0.325 | 1.065 | 0.046 | 24.784 | 21.987 | 0.961 | 0.094 | 0.562 | 0.013 |

| SLNO | GROUPS | MEAN  Y1 | DEVI Y1 | MEAN Y2 | DEVI Y2 | MEAN Y3 | DEVI Y3 | MEAN Y4 | DEVI Y4 | MEAN Y5 | DEVI Y5 | MEAN Y6 | DEVI Y6 |
| --- | --- | --- | --- | --- | --- | --- | --- | --- | --- | --- | --- | --- | --- |
| 1 | 1 | 14.942 | 0.09 | 14.831 | 0.099 | 0.0622 | 0.185 | 0.142 | 0.261 | 0.121 | 0.284 | 14.174 | 0.2599 |
| 2 | 1 | 7.334 | 7.334 | 7.334 | 7.334 | 7.334 | 7.334 | 7.334 | 7.334 | 7.334 | 7.334 | 7.334 | 7.334 |
| 3 | 1 | 14.892 | 0.105 | 14.982 | 0.211 | 0.091 | 0.191 | 0.191 | 0.321 | 0.229 | 0.296 | 14.273 | 0.286 |
| 4 | 1 | 14.159 | 0.087 | 14.529 | 0.087 | 0.051 | 0.181 | 0.131 | 0.211 | 0.085 | 0.193 | 13.983 | 0.041 |
| 5 | 1 | 12.123 | 0.045 | 14.236 | 0.063 | 0.052 | 0.274 | 0.141 | 0.256 | 0.089 | 0.159 | 13.589 | 0.025 |
| 6 | 1 | 14.321 | 0.652 | 14.325 | 0.035 | 0.055 | 0.981 | 0.159 | 0.652 | 0.087 | 0.951 | 14.652 | 0.048 |
| 7 | 1 | 14.652 | 0.362 | 14.625 | 0.058 | 0.089 | 0.685 | 0.952 | 0.369 | 0.081 | 0.156 | 12.695 | 0.049 |
| 8 | 1 | 14.256 | 0.523 | 14.325 | 0.068 | 0.078 | 0.356 | 0.256 | 0.963 | 0.083 | 0.659 | 13.657 | 0.037 |
| 9 | 1 | 12.265 | 0.365 | 12.458 | 0.035 | 0.045 | 0.658 | 0.987 | 0.784 | 0.082 | 0.746 | 14.695 | 0.067 |
| 10 | 1 | 14.325 | 0.785 | 11.326 | 0.012 | 0.089 | 0.785 | 0.856 | 0.859 | 0.085 | 0.631 | 14.778 | 0.088 |

| SLNO | GROUPS | MEAN  Y1 | DEVI Y1 | MEAN Y2 | DEVI Y2 | MEAN Y3 | DEVI Y3 | MEAN Y4 | DEVI Y4 | MEAN Y5 | DEVI Y5 | MEAN Y6 | DEVI Y6 |
| --- | --- | --- | --- | --- | --- | --- | --- | --- | --- | --- | --- | --- | --- |
| 1 | 2 | 7.334 | 7.334 | 7.334 | 7.334 | 7.334 | 7.334 | 7.334 | 7.334 | 7.334 | 7.334 | 7.334 | 7.334 |
| 2 | 2 | 0.081 | 0.261 | 14.8 | 0.05 | 0.062 | 0.006 | 0.09 | 6.762 | 0.098 | 6.672 | 7.005 | 7.532 |
| 3 | 2 | 0.005 | 0.018 | 14.15 | 0.046 | 0.012 | 0.005 | 0.105 | 7.672 | 0.98 | 6.43 | 6.673 | 6.543 |
| 4 | 2 | 0.09 | 0.321 | 15.87 | 0.046 | 0.09 | 0.03 | 0.018 | 7.762 | 0.098 | 0.072 | 6.765 | 7.432 |
| 5 | 2 | 0.02 | 0.025 | 13.65 | 0.045 | 0.055 | 0.004 | 0.012 | 7.856 | 0.094 | 0.067 | 6.784 | 7.365 |
| 6 | 2 | 0.063 | 0.032 | 14.652 | 0.043 | 0.013 | 0.012 | 0.056 | 7.869 | 0.092 | 0.068 | 6.326 | 7.852 |
| 7 | 2 | 0.052 | 0.014 | 12.62 | 0.035 | 0.065 | 0.018 | 0.031 | 7.651 | 0.096 | 0.078 | 6.159 | 7.984 |
| 8 | 2 | 0.006 | 0.052 | 12.336 | 0.063 | 0.038 | 0.065 | 0.037 | 7.326 | 0.093 | 0.071 | 6.149 | 7.326 |
| 9 | 2 | 0.033 | 0.062 | 14.332 | 0.052 | 0.045 | 0.095 | 0.089 | 7.315 | 0.098 | 0.069 | 6.339 | 7.965 |
| 10 | 2 | 0.065 | 0.032 | 14.995 | 0.036 | 0.086 | 0.034 | 0.025 | 7.325 | 0.094 | 0.061 | 7.885 | 7.125 |

| SLNO | GROUPS | MEAN  Y1 | DEVI Y1 | MEAN Y2 | DEVI Y2 | MEAN Y3 | DEVI Y3 | MEAN Y4 | DEVI Y4 | MEAN Y5 | DEVI Y5 | MEAN Y6 | DEVI Y6 |
| --- | --- | --- | --- | --- | --- | --- | --- | --- | --- | --- | --- | --- | --- |
| 1 | 3 | 14.892 | 0.105 | 14.982 | 0.211 | 0.091 | 0.191 | 0.191 | 0.321 | 0.229 | 0.296 | 14.273 | 0.286 |
| 2 | 3 | 0.76 | 0.098 | 0.098 | 1.769 | 0.097 | 0.73 | 45.02 | 0.098 | 0.098 | 0.76 | 14.45 | 0.342 |
| 3 | 3 | 0.456 | 10.76 | 0.056 | 34.87 | 0.54 | 0.098 | 34.87 | 0.098 | 0.311 | 0.098 | 14.65 | 0.321 |
| 4 | 3 | 1.54 | 0.098 | 0.65 | 0.098 | 0.087 | 10.87 | 0.098 | 0.076 | 0.65 | 1.098 | 13.43 | 0.123 |
| 5 | 3 | 0.361 | 0.085 | 0.32 | 0.032 | 0.065 | 0.63 | 0.097 | 0.096 | 0.61 | 0.098 | 13.46 | 0.631 |
| 6 | 3 | 0.253 | 0.095 | 0.365 | 0.087 | 0.088 | 0.096 | 0.095 | 0.031 | 0.66 | 0.096 | 14.35 | 0.136 |
| 7 | 3 | 0.365 | 0.065 | 0.012 | 0.058 | 0.097 | 0.035 | 0.096 | 0.34 | 0.64 | 0.095 | 13.65 | 0.316 |
| 8 | 3 | 0.125 | 0.074 | 0.056 | 0.068 | 0.094 | 0.012 | 0.091 | 0.078 | 0.69 | 0.059 | 14.36 | 0.258 |
| 9 | 3 | 0.312 | 0.045 | 0.0481 | 0.098 | 0.044 | 0.0561 | 0.093 | 0.098 | 0.62 | 0.091 | 14.89 | 0.852 |
| 10 | 3 | 0.895 | 95 | 0.0356 | 0.08 | 0.074 | 0.0895 | 0.097 | 0.074 | 0.63 | 0.097 | 13.69 | 0.529 |

| SLNO | GROUPS | MEAN  Y1 | DEVI Y1 | MEAN Y2 | DEVI Y2 | MEAN Y3 | DEVI Y3 | MEAN Y4 | DEVI Y4 | MEAN Y5 | DEVI Y5 | MEAN Y6 | DEVI Y6 |
| --- | --- | --- | --- | --- | --- | --- | --- | --- | --- | --- | --- | --- | --- |
| 1 | 4 | 14.159 | 0.087 | 14.529 | 0.087 | 0.051 | 0.181 | 0.131 | 0.094 | 0.085 | 0.193 | 13.983 | 0.041 |
| 2 | 4 | 0.098 | 0.097 | 14.65 | 0.087 | 0.098 | 0.132 | 0.321 | 0.234 | 0.009 | 0.231 | 12.87 | 0.072 |
| 3 | 4 | 0.176 | 0.067 | 23.78 | 1.98 | 0.089 | 0.321 | 0.323 | 0.432 | 0.076 | 0.021 | 12.78 | 0.123 |
| 4 | 4 | 0.098 | 1.09 | 1.009 | 0.097 | 0.097 | 0.123 | 0.123 | 0.112 | 0.321 | 0.213 | 0.021 | 1.021 |
| 5 | 4 | 0.0642 | 0.062 | 0.362 | 0.096 | 0.062 | 0.965 | 0.451 | 0.558 | 0.312 | 0.456 | 0.0297 | 1.036 |
| 6 | 4 | 0.523 | 0.045 | 0.12 | 0.069 | 0.68 | 0.659 | 0.652 | 0.334 | 0.784 | 0.956 | 0.0315 | 1.036 |
| 7 | 4 | 0.658 | 0.036 | 0.3665 | 0.086 | 0.096 | 0.785 | 0.912 | 0.477 | 0.951 | 0.784 | 0.0169 | 1.098 |
| 8 | 4 | 0.985 | 0.028 | 0.215 | 0.097 | 0.078 | 0.852 | 0.219 | 0.688 | 0.961 | 0.684 | 0.0569 | 1.037 |
| 9 | 4 | 0.785 | 0.098 | 0.365 | 0.091 | 0.036 | 0.891 | 0.129 | 0.356 | 0.894 | 0.651 | 0.0659 | 1.098 |
| 10 | 4 | 0.654 | 0.075 | 0.852 | 0.093 | 0.075 | 0.678 | 0.631 | 0.971 | 0.885 | 0.358 | 0.0169 | 1.098 |
